# Adolescents, Graduated Autonomy, and Genetic Testing

**DOI:** 10.1155/2012/946032

**Published:** 2012-02-23

**Authors:** Susan Fox

**Affiliations:** 152 Paradise Rd., Golden, Colorado 80401, USA

## Abstract

Autonomy takes many shapes. The concept of “graduated autonomy” is conceived as comprising several unique features: (1) it is incremental, (2) it is proportional, and (3) it is related to the *telos* of the life stage during which it occurs. This paper focuses on graduated autonomy in the context of genetic testing during adolescence. Questions can be raised about other life stages as well, and some of these questions will be addressed by discussing a possible fourth characteristic of graduated autonomy, that is, its elasticity. Further scholarship and analysis is needed to refine the concept of graduated autonomy and examine its applications.

*“There is no steady. . . progress in this life; we do not advance through fixed gradations, and at the last one pause through infancy’s unconscious spell, boyhood’s thoughtless faith, adolescence’ doubt (the common doom), then skepticism, then disbelief, resting at last in manhood’s pondering repose of If. But once gone through, we trace the round again; and are infants, boys, and men, and Ifs eternally. Where lies the final harbor, whence we unmoor no more?”*

Herman Melville

## 1. Introduction

Spinning, weaving, and severing the threads of life, three divine sisters known as “the Fates” were held accountable for human destiny in the era of Greek mythology. Millennia later, in the era of chromosome testing, we are still learning how genetic fibers contribute to life's intricate tapestry. Patterns, of course, are not determined by threads alone. To a greater or lesser degree, we exercise autonomy and assume moral responsibility for choosing our goals and charting our course. We can even decide how familiar we want to become, through DNA testing, with our own genetic material.

Autonomy is a means of inspiring choice and guiding decisions, but it is not, I submit, an all-or-nothing proposition. Drawing upon various developmental theories, this paper introduces the concept of graduated autonomy, identifies its core elements, and explores its application to adolescent genetic testing. It then reviews genetic testing recommendations by various professional and political bodies and compares them for consistency with criteria for graduated autonomy.

Three preliminary observations are in order. First, this discussion is limited to genetic testing and does not embrace genetic research or genomic therapy. Second, issues of genetic testing, autonomy, and children's rights are global in scope. Principles, position papers, and pronouncements have been issued by national, supranational, and international agencies including the American Pediatric Society, the European Union, and the United Nations. Third, it should not be assumed that exercising autonomy always leads to action. To the contrary, the decision to refrain from genetic testing or avoid learning its results can involve the same degree of autonomy as the decision to undertake testing and embrace its outcome. “Autonomy includes both the right to know and the right not to know one's genetic status” [1]. In an imperfect world, limited space constrains our ability to address many seemingly limitless questions. This paper, then, is merely a first step in a new direction.

## 2. Autonomy Examined

In order to take a “graduated” approach to autonomy, we must try to understand what the term autonomy means in the first place. Derived from the Greek “auto” (self) and “nomos” (law), autonomy has been defined succinctly as self-governance. This does not answer the question, however, of whether autonomy is a state of being, or a process, or both.

Autonomy has been described as the ability to regulate one's own behavior [1]. One might regard autonomy as a status or an achievement, for example, “*When I reach maturity, I attain autonomy*.” From this perspective, autonomy is a state of being. Autonomy in adolescence is not necessarily a unitary concept, however [2]. According to social domain theory, “youth may achieve different levels of decision-making autonomy depending on the domain under which the decision falls” [3]. Various domains of autonomy involve personal, social, cognitive, and behavioral aspects [3]. From this perspective, autonomy can be viewed as a whole which is greater than the sum of its parts.

From other perspectives, autonomy is a dynamic, adaptive work-in-progress, for example, “*As I mature through my teen-age years, I become more autonomous.*” During adolescence, the task of “becoming” autonomous involves distancing and individuating from one's parents and taking responsibility for oneself. This does not necessarily require severing familial ties, but renegotiating them. “Once regarded as the process of striving to gain freedom from one's parents, autonomy… is now understood as an interpersonal process by which the adolescent begins to develop a greater capacity for independent behavior in the context of continued family connections” [4]. In broader terms, autonomy has been referred to as the process of becoming a self-governing person [5]. “(I)n contrast to traditional conceptualizations of adolescence as a time of breaking the parent-child bond, recent evidence supports a view of this period as one of gradual renegotiation between parents and children from the asymmetrical authority of early and middle childhood toward, potentially, a peer-like mutuality in adulthood” [6].

In both iterations, as a state of being *or* as a process, autonomy concerns our relational dimension. There is both freedom *from* an “other” and freedom* to* express the “self.” As observed by Soenens and colleagues, adolescent autonomy encompasses two separate (but often simultaneous) phenomena: separation and individuation from parents and self-actualization or “self-determination” [7]. The former occurs as adolescents physically and emotionally distance themselves from their parents (separation) and increasingly take responsibility for themselves (individuation). The latter concentrates on “the degree to which behaviors are enacted with a sense of volition” and authentic “choicefulness” [7]. In terms of separation-individuation theory, the opposite of autonomy is dependence. In terms of self-actualization, “the opposite of autonomy is not dependence but heteronomy, that is, the feeling of being controlled… by external forces or by internal compulsions” [7].

A third view of autonomy might regard it as neither a static condition nor a fluid process, but rather as a competency. In this sense, autonomy can be likened to a tool or a skillset, which can be “disaggregated” into cognitive, emotional, and behavioral features [4]. The seeds of graduated autonomy lie in this more nuanced approach. Graduated autonomy takes root—or loses ground—in intervals (the “incremental” element). It proceeds in scale (the “proportional” element) to involve the individual's other characteristics in light of the momentum of one's stage in life (the *telos*) where graduated autonomy is being engaged.

It has long been acknowledged that becoming autonomous is a central developmental task for adolescents [3]. This is not to say, however, that adolescence is the *only* time during a life cycle when autonomy develops. Indeed, one thesis of this paper is that autonomy during adolescence can properly be viewed as a substrate of graduated autonomy throughout life.

## 3. Aspects of Graduated Autonomy

### 3.1. Theoretical Basis

From a developmental perspective, human growth moves through sequential phases of cognitive, emotional, behavioral, and moral awareness during childhood and adolescence. Piaget, Kohlberg, Gilligan, and Erikson have all contributed to our understanding of character formation during life's first two decades. The persistent challenge has been that a linear conception of these phases does not completely explain the uneven rate at which adolescents move through them.

In the realm of genetic research, concepts of assent and joint decision-making have been adopted to fill in the gaps. As Miller and Nelson explain “Joint decision-making between parents and children typically precedes full decision-making autonomy during adolescence… A developmental approach to assent would respect that shared decision-making between parents and children is a characteristic of normal development” [8].

From the standpoint of adolescent psychodynamics, there may be at least one critical difference between genetic research and genetic testing. Testing is directed at the personal architecture of an individual subject, whereas research focuses on a larger demographic wall in which the individual subject is merely one brick. Vulnerability may be experienced much differently during testing than it is during research, and yet again differently depending upon whether testing arises from individual or family motivations. In either case, vulnerability is a cornerstone principle in European bioethics and its implications for genetic testing invite further scrutiny.

### 3.2. The Relationship between Graduated Autonomy and Assent

Assent in the context of genetic research and graduated autonomy in the realm of genetic testing share several common elements. First, assent is premised (in part) on assumptions that today's assenting minor is likely to become tomorrow's consenting adult.

“(S)olicitation of assent from minors engages them in graduated levels of decision-making, in which they participate in developmentally appropriate ways. Assent thus serves as a “learner”s permit' for decision-making, enabling adolescents to gradually assume independence so that full decision-making autonomy is not exercised until they have some experience with the task” [9].

In this regard, assent and graduated autonomy both operate on a continuum, reflecting a common incremental approach.

In the second place, assent is tinged with proportionality. In a thoughtful discussion of relationships between physicians and their younger patients, the American Pediatric Association observes that assent can “empower children to the extent of their capacity” and counsels pediatricians to “give serious consideration to each patient's developing capacities for participating in decision-making, including rationality and autonomy” [10].

In other words, the benefit that minor patients derive from assent is scaled to the degree that they can participate in the process of giving it.

Notwithstanding these correlations, however, graduated autonomy (in the context of genetic testing) is not merely the functional equivalent of assent (in the realm of genetic research). Assent is a precursor to agreement [11]. Graduated autonomy, on the other hand, can be a prelude to either assent or refusal. It is preliminary to the process of deciding whether to agree *or not *and can be viewed as a condition during which individual capabilities are crystallizing (or dissolving).

When viewed in the larger context of the full human life cycle, graduated autonomy can be conceived as the foundation of assent and joint decision-making during adolescence *as well as* assisted decision-making during advanced years. First visible through the topsoil of childhood, graduated autonomy becomes alloyed with age and maturity. It is melded into one of adulthood's supporting girders, and then as cognitive, emotional, and social layers of selfhood erode, it reappears as a residue of self-hood. Metaphorically, it can be compared to the lifecycle of a mosaic design in which numerous tiles are selected, assembled, cemented, and then worn away over time.

The broader philosophical, psychiatric, and behavioral implications of graduated autonomy across the age spectrum remain open to debate and examination in future works. For present purposes, we look to the incremental, proportional, and teleological aspects of graduated autonomy within the narrower context of genetic testing decisions for adolescents.

### 3.3. Graduated Autonomy as Incremental

In an effort to identify optimum times during teen-age years for exercising autonomy regarding genetic testing, Wehbe and colleagues conducted in-depth interviews of adolescent females at risk for being carriers of fragile X syndrome [12]. The study, conducted between 2003 and 2006, included 53 adolescent girls and young women from 13 different states in the USA. The investigators examined several phases of awareness about the condition including knowledge about genetic inheritance as a concept, knowledge about possible carrier status, and knowledge confirming carrier status based on genetic testing.

Results indicated that first-stage awareness, that is, general awareness about the inherited nature of certain diseases, was regarded as being appropriate to be introduced at younger ages than actual knowledge about carrier status.

“The majority of the adolescent and young adults in this study felt that a child should learn that fragile X syndrome is an inherited disorder in early childhood and no later than the preteen years. Participants felt that it was important to learn this information early, often endorsing staging of the information in a developmentally appropriate manner” [12].

According to Wehbe's study, the optimum time for second-stage awareness, that is, the possibility of carrier status, is during preteen and teen-age years.

“Study participants felt that it was important for a girl to be old enough to have the necessary level of intellectual maturity to understand the implications of what “being a carrier…” means for future reproduction. Many were concerned that a younger child might not be able to understand this information. Additionally, some participants felt that information regarding risk status should be timed concurrently with physical maturity, expressing concerns for an unplanned pregnancy and/or reproductive decision-making during this time” [12].

As voiced by one participant, the teen-age years are appropriate for disclosure of potential carrier status “because I mean you can actually understand it, and it's more known to you, like, you realize what it means and stuff” [12].

Wehbe's results are consistent with findings among adolescents at risk for carrying other types of genetic disease. Järvinen et al., surveyed young adult women with a family history of Duchenne muscular dystrophy and hemophilia A. They reported that the majority of subjects believed that carrier testing should occur during childhood or teen-age years [13].

On the other hand, some teen-agers prefer to wait until later before knowing results of genetic tests. James and colleagues studied nine adolescent females who each had a sibling affected by chronic granulomatous disease and discovered that “many of these girls felt carrier testing for this disorder should not be offered until 18 years of age or older” [14].

In another study illustrating the incremental features of graduated autonomy, Wray-Lake and colleagues mapped trajectories of autonomous decision-making over ten years in 201 American families [3]. Their findings revealed that in most instances, autonomy increased gradually during middle school, early, and mid-adolescence, and then rose precipitously during late adolescence. This pattern did not apply to health care decisions, which progressed in a steady linear fashion across all ages. Nonetheless, all trajectories advanced in discernible intervals.

Although ages may vary, it remains true that “(f)or children and adolescents, decision-making autonomy develops gradually and involves taking on increased responsibility for making decisions previously made by parents” [8]. All of these studies illustrate the incremental nature of graduated autonomy, advancing in progressive steps through adolescence.

### 3.4. Graduated Autonomy as Proportional

In the context of graduated autonomy, proportionality means “scaled to life conditions.” For example, research indicates that autonomy occurs differently for adolescents with spina bifida than it does for teenagers without inherited disease, but there is a rough symmetry between the curves. Adolescents with spina bifida, compared to each other, also mature at different rates in proportion to the burdens of their disabilities.

These findings came to light when Friedman and colleagues conducted an eight-year longitudinal study of some 68 children and adolescents, ages 9 through 15, who were affected by spina bifida [4]. Investigators charted autonomy growth curves for these youth and compared them to autonomy curves generated by an equivalent number of subjects at the same ages without spina bifida. Results were evaluated in terms of dependent behavior, motivation, and emotional autonomy.

Researchers predicted that children affected by spina bifida “would lag behind their peers in autonomy development, starting at a lower level of autonomy and additionally demonstrating a less rapid growth trajectory as they transition into adolescence” [4]. Results yielded a “somewhat more complicated picture of autonomy… within the context of a chronic illness,” but supported the conclusion that “group differences between children with spina bifida and their age-matched peers in developmental trajectories were evident across measures of autonomy” [4]. There was imperfect but obvious symmetry between growth curves of the control group and those of spina bifida teenagers. The results were roughly (although not always) parallel. The investigators concluded that their findings provided evidence that “children with spina bifida demonstrate distinct developmental trajectories across the transition to adolescence with regard to growth in autonomy,” when compared to their healthy peers [4].

Proportionality in the context of autonomy can also be based on comparative maturity. Speaking on behalf of the European Society for Human Genetics, Borry and colleagues recommend that “in the context of genetic testing, the opinion of minors should be taken into consideration as an increasingly determining factor in proportion to his or her age and degree of maturity” [15, 16]. According to Borry et al., maturity is a more significant marker for autonomy than age:

“There is a huge individual and societal variation regarding the moment when particular levels of competence are achieved. As a consequence from an ethical perspective, a rule about competence that is solely based on age cannot be satisfactory… All children do not develop in the same way. Children of the same age may have a different level of development or maturity. As soon as minors, in proportion to their age and degree of maturity, are able to participate in the decision-making, their opinion should be taken increasingly into consideration” [15, 16].

In effect, Borry et al. recommend that the weight given to an adolescent's opinion about genetic testing should be gauged in proportion to the maturity of the decision-maker. The authors also mention a number of other important factors that should be considered:

“When assessing competence, it is important not to assess general competence, but to assess a patient's level of understanding in relation to a specific choice that has to be made. “The nature and complexity of the decision or task, the person's ability to understand, at the time the decision is made, the nature of the decision required, and its implications are all relevant. Thus, the graver the impact of the decisions, the commensurately greater the competence needed to make it” [15, 16].

In other words, proportionality should consider the type of disease, the gravity of the decision, *and *the maturity of the patient.

### 3.5. Teleological Aspects of Graduated Autonomy

An additional theoretical basis exists for graduated autonomy, grounded in teleology. In rough terms, the *telos* or goal of adolescence is maturity. Hence, as autonomy develops during adolescence, it can be conceived as progressing towards an end, namely, adulthood. In this sense, graduated autonomy in adolescence evolves toward mature autonomy, as youth is driven to age.

Miller and Nelson suggest that assent is a springboard to full-blown informed consent and is teleological in that sense as well.

“Joint decision-making between parents and children typically precedes full decision-making autonomy during adolescence. The concept of joint decision-making… serves as a useful framework from which to understand the way in which child assent can facilitate children's growing autonomy. In addition, this (developmental) framework is potentially useful for understanding the process by which adolescents become fully autonomous and capable decision-makers themselves” [8].

This should *not* be interpreted to mean that graduated autonomy occurs *only* during adolescence, however. To the contrary, extrapolating from statistical patterns in their study of 153 Flemish adolescents in Belgium, Soenens, and colleagues concluded that there is an “innate need for autonomy that is essential for optimum functioning across the life span” [7].

An intuitive basis exists for positing that graduated autonomy (like life itself) could proceed along a parabolic curve, advancing through adolescence into adulthood until, in keeping with the *telos* of human aging, it incrementally declines. “(T)hroughout life, autonomy advances and declines as individuals develop new competencies and changing conditions require alterations in behavior” [17].

It follows, then, that graduated autonomy could wax *or *wane. Another way to frame this corollary is that autonomy can be graduated in either direction. (e.g., a once-competent patient “fades away” into advancing dementia.) In this sense, graduated autonomy can be described as “elastic.”

Empirical evidence of this proposition would be difficult to obtain, and it lies beyond the scope of this paper to design appropriate studies. At this point, it can be said only that the application of graduated autonomy applies to life's ebbs, as well as its flows, bears further attention.

## 4. Elements of Graduated Autonomy in Professional Standards and Political Conventions

Graduated autonomy, particularly with respect to its proportionality component, can be a useful heuristic tool for interpreting international health care compacts about health care rights for younger patients.

Article V of the United Nations Convention on the Rights of the Child recognizes that parents and adult caretakers have rights, duties, and responsibilities for furnishing a child with “appropriate” direction and guidance “in a manner consistent with the evolving capacities of the child” [18]. Article XII of the Convention charges signatory parties with responsibility to “assure” that a minor who is capable of forming individual views has the right to express those views freely, with “due weight… being given… in accordance with the age and maturity of the child” [18]. Both Articles thus employ proportionality as a means of gauging “appropriate” conduct or “due weight.”

Graduated autonomy, correlated with proportionality, is also manifest in the European Convention on Human Rights and Biomedicine adopted by the Council of Europe. Article 6 of the Convention relates to the protection of persons (including minors) who are not capable of full informed consent. Paragraph 2 of Article 6 stipulates that “where according to law, a minor does not have the capacity to consent to an intervention… the opinion of the minor shall be taken into consideration as an increasingly determining factor, in proportion to his or her age and degree of maturity” [19].

Another facet of graduated autonomy's heuristic value, when interpreting professional norms, can be found in its incrementalism. The American Academy of Pediatrics has declared that “as children develop, they should gradually become the primary guardians of personal health and the primary partners in medical decision-making, assuming responsibility from their parents” [10]. The inclusion of the word “gradually” cannot be assumed to be accidental or casual. It coincides closely with the sense of incremental progress characterized by graduated autonomy.

## 5. Conclusion

Given the sensitivity and volatility of family relationships during teen-age years, decisions about genetic testing deserve careful attention. To focus only on test results would overlook a crucial ingredient in the mélange of adolescence: the choice about whether to engage in the act of testing indicates, *per se*, an appetite for identity. For some adolescents, a decision to avoid testing signals their belief that personal identity is not determined by reference to genetic lineage. For others, a choice to undergo testing announces their acceptance of genetic influence. In either case, a decision to test (or not) is a declaration of self-awareness.

The concept of graduated autonomy is a framework for understanding decisions by adolescents about genetic testing. Considerable research and reflection will be needed to further refine graduated autonomy and its elements of incrementalism, proportionality, elasticity, and correlation to the *telos* of life stages. Conversations and colloquia involving developmental psychologists, child and adolescent psychiatrists, and genetic counselors can make valuable contributions, along with participation from pediatricians and philosophers.

We walk upon the carpet of the past when approaching the threshold of the future, and many strands of thought entwine with our genetic fibers to weave a tapestry of possibilities. Graduated autonomy, it is hoped, adds a helpful thread to these patterns.
